# Supplementary material for: Use of A Hydroalcoholic Extract of Moringa oleifera Leaves for the Green Synthesis of Bismuth Nanoparticles and Evaluation of Their Anti-Microbial and Antioxidant Activities
Source: Materials (Basel). 2020 Feb 15;13(4):876. doi: 10.3390/ma13040876 (PMC7079629; doi:10.3390/ma13040876)
Supplement: Supplementary file 1 [file materials-13-00876-s001.pdf]

materials

MDPI

Supplementary Materials: Use of A Hydroalcoholic Extract of *Moringa oleifera* Leaves for the Green Synthesis of Bismuth Nanoparticles and Evaluation of their Anti-microbial and Antioxidant Activities

Prince Edwin Das [1], Amin F. Majdalawieh [2,*], Imad A. Abu-Yousef [2], Srinivasan Narasimhan [1,*], and Palmiro Poltronieri [3,*]

[1] Asthagiri Herbal Research Foundation, 162A, Perungudi Industrial Estate, Perungudi, Chennai, India 600096; prince.ahrf@gmail.com

[2] Department of Biology, Chemistry and Environmental Sciences, American University of Sharjah, P.O. Box 26666, Sharjah, United Arab Emirates; iabuyousef@aus.edu

[3] Institute of Sciences of Food Productions, CNR-ISPA, Lecce 73100, Italy

* Correspondence: amajdalawieh@aus.edu (A.F.M.); narasimhan_s@yahoo.com (S.N.); palmiro.poltronieri@ispa.cnr.it (P.P.); Tel.: (971) 6 5152429 (A.F.M.); (91) 44 22397645 (S.N.); Fax: (971) 6 515 2450 (A.F.M.); (91) 44 22397645 (S.N.).

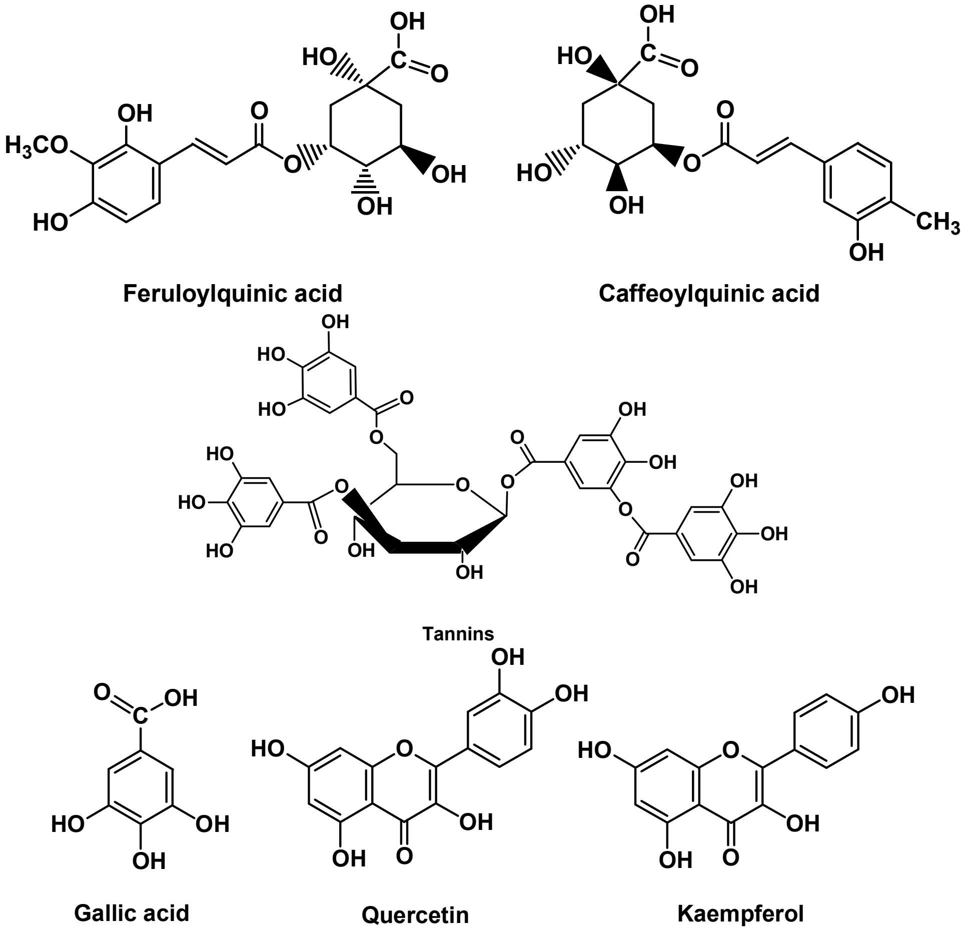

Figure S1. Chemical structures of caffeoylquinic acid, feruloylquinic acid, tannins, gallic acid, quercetin, kaempferol.

© 2020 by the authors. Licensee MDPI, Basel, Switzerland. This article is an open access article distributed under the terms and conditions of the Creative Commons Attribution (CC BY) license (http://creativecommons.org/licenses/by/4.0/).
